# The dialog between mother and newborn: insights from immune mediator crosstalk elicited by antenatal SARS-COV-2 exposure

**DOI:** 10.3389/fimmu.2025.1606582

**Published:** 2025-07-31

**Authors:** Maria Eduarda Canellas-de-Castro, Lizandra Moura Paravidine Sasaki, Geraldo Magela Fernandes, Felipe Motta, David Alves de Araújo Júnior, Heidi Luise Schulte, Ângelo Pereira da Silva, Caroline de Oliveira Alves, Rosana Maria Tristão, José Alfredo Lacerda de Jesus, Karina Nascimento Costa, Luiz Claudio Gonçalves de Castro, Otávio de Toledo Nóbrega, Laila Salmen Espindola, Jordana Grazziela Alves Coelho-dos-Reis, Joaquim Pedro Brito-de-Sousa, Ismael Artur da Costa-Rocha, Vitor Hugo Simões Miranda, Ana Carolina Campi-Azevedo, Vanessa Peruhype-Magalhães, Andréa Teixeira-Carvalho, Ciro Martins Gomes, Alberto Carlos Moreno Zaconeta, Cleandro Pires de Albuquerque, Licia Maria Henrique da Mota, Olindo Assis Martins-Filho, Alexandre Anderson de Sousa Munhoz Soares

**Affiliations:** ^1^ Programa de Pós-Graduação em Ciências Médicas, Universidade de Brasília (UnB), Brasília, Brazil; ^2^ Hospital Universitário de Brasília, Universidade de Brasília (UnB), Brasília, Brazil; ^3^ Faculdade de Medicina, Universidade de Brasília (UnB), Brasília, Brazil; ^4^ Neonatologia do Hospital Regional de Sobradinho - Secretaria de Saúde do Distrito Federal (SES/DF), Brasilia, Brazil; ^5^ Laboratório de Virologia Básica e Aplicada, Instituto de Ciências Biológicas, Universidade Federal de Minas Gerais, Belo Horizonte, Brazil; ^6^ Instituto René Rachou, Fundação Oswaldo Cruz (FIOCRUZ-Minas), Belo Horizonte, Brazil; ^7^ Programa de Pós-Graduação em Patologia Molecular, Universidade de Brasília (UnB), Brasília, Brazil

**Keywords:** COVID-19, maternal-fetal communication, immunological mediators, umbilical cord blood, maternal serum, prenatal exposure

## Abstract

**Goal:**

The present study intended to evaluate whether the profile of soluble immune mediators observed in cord blood samples resembles the pattern identified for mother serum samples.

**Methods:**

For this purpose, parallel analysis of chemokines, cytokines, and growth factors was carried out in mother–newborn paired samples from acute and convalescent COVID-19 subgroups (Early, Intermediate, and Late) as well as healthy controls (HC).

**Results:**

Data demonstrated that increased levels of CCL11, IFN-γ, IL1-Ra, and G-CSF were observed in cord blood samples from most COVID-19 subgroups, with fold change magnitude from 1.6× to 8.2× as compared with HC. Comparative analysis of mother–newborn pairs demonstrated that several immune mediators (CCL11, CCL4, IFN-γ, PDFG, and G-CSF) exhibited high increment magnitude in cord blood as compared with mother serum, reaching values up to 15.7×, mainly at convalescent COVID-19 infection. The signatures of soluble immune mediators revealed distinct waveforms for cord blood and mother serum, with a waning of immune mediators in the latter, contrasting with the increasing set of molecules in the former from acute toward convalescent COVID-19. Integrative network analysis of immune mediators in mother–newborn pairs showed an increase of neighborhood connectivity both in microenvironments and in their interplay from acute toward late convalescent COVID-19. Our results support the hypothesis of the interplay between mother serum and cord blood microenvironment that may impact the fetus development.

**Conclusion:**

Together, this evidence regarding the maternal–fetal crosstalk can ultimately subsidize the improvement of clinical practice and public health policies for management of prenatal exposure to SARS-CoV-2 infection.

## Introduction

Since declared a global pandemic in, 2020, the COVID-19 caused by the SARS-CoV-2 virus has significantly disrupted the global public health systems, representing unique challenges to vulnerable populations such as children, elderly, and pregnant women (1, 2).

Although the frequency of vertical transmission of SARS-CoV-2 is still debatable, previous studies have provided evidence that viral infection during pregnancy leads to several intrauterine and fetal effects of transplacental transfer of soluble immune mediators, including virus-specific antibodies and cytokines, in response to maternal infections, including SARS-CoV-2 (3–5).

Infections during pregnancy not only pose a risk to maternal health but also have potential implications for the fetal development (6–8). The maternal immune system transition during pregnancy from a pro-inflammatory state at pregnancy onset to an anti-inflammatory profile toward mid-late pregnancy stages can contribute to higher vulnerability to infections (9, 10). The dynamic interplay between pro-inflammatory and anti-inflammatory immunological responses that occur at distinct gestational phases is crucial to safeguarding maternal health and ensuring a successful gestation (11).

The study of immune mediator profiles in maternal and neonatal contexts has garnered significant attention due to its implications for understanding the prenatal immune environment and fetal outcomes elicited by maternal–fetal immune interaction (12). The immunological changes during pregnancy have significant implications for the soluble mediator networks at the maternal–fetal interface orchestrating the fetal development and newborn health (13). The SARS-CoV-2 infection during pregnancy poses increased risks of adverse maternal and newborn outcomes (14).

Understanding the dynamics between maternal infection and fetal immune responses is crucial for assessing how prenatal exposure to SARS-CoV-2 might affect children health. The comprehensive impact of SARS-CoV-2 infection on the maternal–fetal immune interface is still poorly understood. In this line, the present study aimed to investigate the changes of soluble immune mediators in mothers with acute or convalescent COVID-19 and their impact in the umbilical cord blood immunological microenvironment. Our findings provided evidence regarding the maternal–fetal crosstalk that can ultimately subsidize the improvement of clinical practice and public health policies for management of prenatal exposure to SARS-CoV-2 infection.

## Population, materials and methods

### Study population

This study is part of a larger prospective observational investigation, named PROUDEST Project (Pregnancy Outcomes and Child Development Effects of SARS-CoV-2 Infection Study) (15), carried out between July, 2020 and December, 2021 in the Federal District of Brazil, during the circulation of the B.1.1.28 and B.1.1.33 SARS-COV-2 variants. The study protocol was submitted and approved by the ethical committee at Faculdade de Medicina, Universidade de Brasília, and by the National Ethical Committee - CONEP (CAAE, 32359620.0.0000.5558) and registered at the Brazilian Clinical Trials Platform – REBEC (https://ensaiosclinicos.gov.br/rg/RBR-65qxs2). The study has followed the ethical principles stated by the Helsinki Declaration for research involving human beings. A total of 149 parturients were invited to participate in this cross-sectional study, as a non-probability convenience sampling from two Public Reference Centers for COVID-19 (Hospital Universitário de Brasília and Hospital Regional da Asa Norte, Brasília, DF, Brazil). All parturients, hereafter referred as “mother,” have provided a written informed consent prior inclusion in the study. The study population comprised paired samples of mother peripheral blood (n=149) and umbilical cord blood from their offsprings (n=149), collected at delivery. The study population was categorized into five groups, based on the mother status for COVID-19, referred to as (i) “Acute”—acute SARS-CoV-2 infection (up to 14 days of symptoms onset at delivery); (ii) “Early”—convalescent SARS-CoV-2 infection acquired at the third pregnancy trimester (28 to 41 weeks); (iii) “Intermediate”—convalescent SARS-CoV-2 infection acquired at the second pregnancy trimester (14 to 27 weeks); (iv) “Late”—convalescent SARS-CoV-2 infection acquired at the first pregnancy trimester (4 to 13 weeks); and (v) “HC”—reference control group comprising non-infected healthy subjects with no clinical history of COVID-19, with negative serology for SARS-CoV-2 infection at delivery and no further diagnosis confirmed up to 30 days after delivery. The exclusion criteria are as follows: mothers vaccinated for COVID-19 before or during pregnancy, and mothers with confirmed diagnosis of toxoplasmosis, syphilis, rubella, herpes, Chagas disease, cytomegalovirus, Zika virus, or human immunodeficiency virus during pregnancy. In addition, smokers and excessive alcohol/illicit drug users were also excluded.

The COVID-19 diagnosis was based on at least one of the following criteria: (a) positive serology for anti-SARS-CoV-2 IgM or IgG by rapid test (Biomanguinhos, Fiocruz, Brazil); (b) positive SARS-CoV-2 quantitative real-time polymerase chain reaction (qRT-PCR) on nasopharyngeal swab specimens; or (c) clinical symptoms suggestive of COVID-19 and a chest computed tomography compatible with COVID 19.

**Figure 1:**
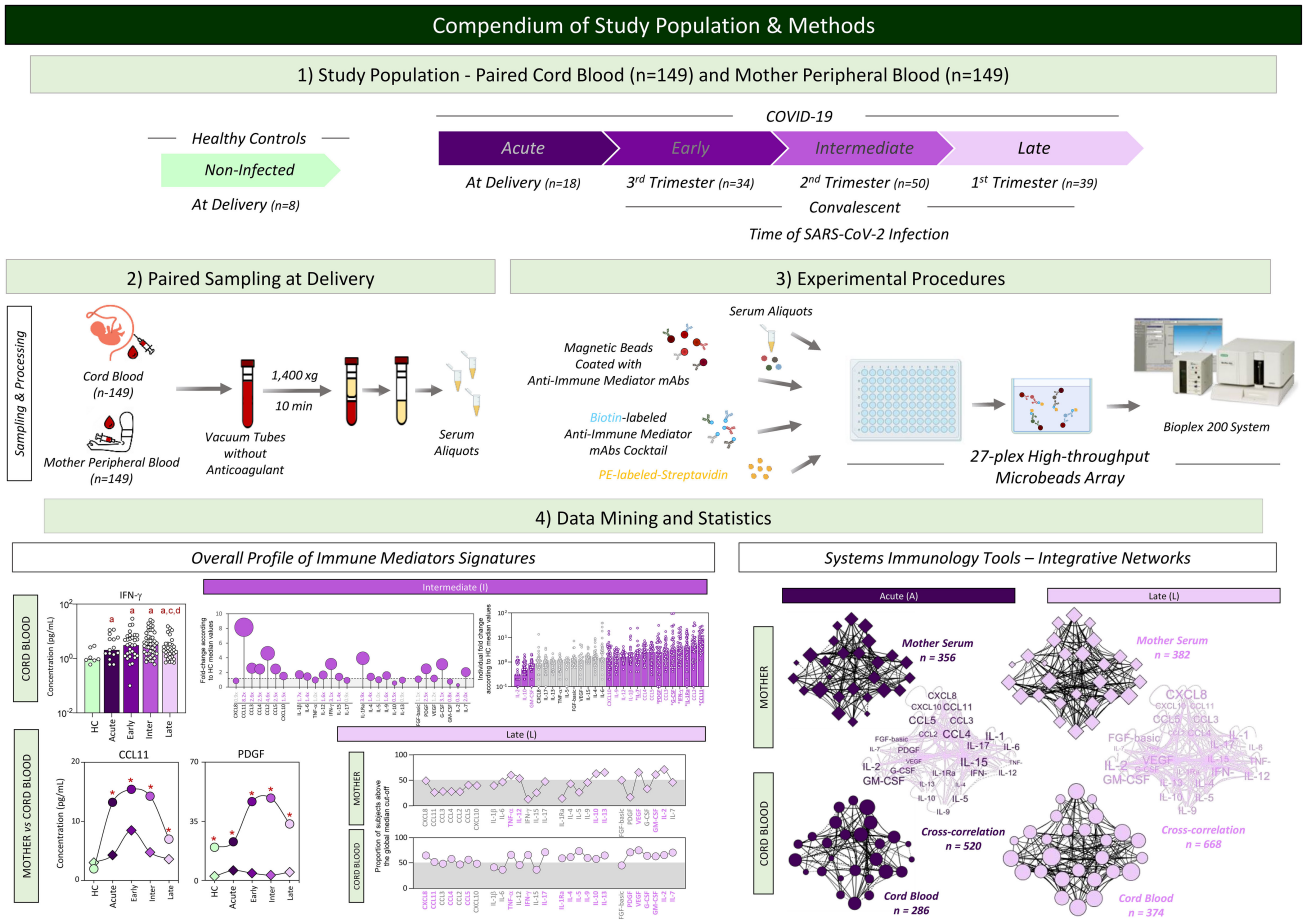
Compendium of study population and methods. This is an exploratory observational investigation enrolling a total of 298 participants to evaluate soluble immune mediators in umbilical cord blood from neonates and paired peripheral blood from mothers with acute or convalescent COVID-19. Acute COVID-19 (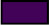, n=18) was defined as SARS-CoV-2 infection up to 14 days upon symptom onset. Convalescent COVID-19 comprising three stages of infection, referred to as (i) Early (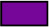, n=34) = previous SARS-CoV-2 infection acquired at the third pregnancy trimester; (ii) Intermediate (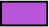, n=50) = previous SARS-CoV-2 infection acquired at the second pregnancy trimester; and (iii) Late (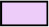, n=39) = previous SARS-CoV-2 infection acquired at the first pregnancy trimester. Healthy Controls, referred to as HC (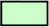, n=8), included non-infected participants. Heparinized plasma samples from umbilical cord and mother peripheral blood were used for experimental procedures to quantify serum immune mediators using 27-plex high-throughput microbead array. Distinct approaches were employed for data mining and statistics, including multiple comparisons of serum immune mediators in cord blood samples from HC and COVID-19 subgroups; fold changes in COVID-19 subgroups according to HC median values; and comparative analysis of serum immune mediator levels and signatures of umbilical cord and mother peripheral blood from HC and COVID-19 subgroups. Systems immunology tools were employed to assemble integrative networks of serum immune mediators in umbilical cord and mother peripheral blood from HC and COVID-19 subgroups as well as in the cord blood/mother cross-correlation throughout the acute and convalescent COVID-19 infection.

A compendium of the study population and methods is provided in **Figure 1**.

### Serum and umbilical cord blood processing and storage

Peripheral blood samples from a total of 149 mothers were collected by venipuncture, at delivery, using vacuum tubes without anticoagulant (HC, n=8; Acute, n=18; Early, n=34; Intermediate, n=50; and Late, n=39). A total of 149 newborn venous cord blood paired samples (5 mL) were collected from the placental part, immediately after clamping using vacuum tubes without anticoagulant. Mother peripheral blood and cord blood specimens were submitted to centrifugation at 1,400 × g, 10 min, 4°C within 6 h after collection, aliquoted, and stored at −80°C until processing for quantification of soluble immune mediators.

### Quantification of soluble immune mediators

The levels of soluble immune mediators were quantified in cord blood and mother serum samples by high-throughput microbead multiplex assay (Bio-Plex Pro™ Human Cytokine 27-plex Assay, Bio-Rad Laboratories, Hercules, CA, USA), according to the manufacturer’s instructions. The concentrations of chemokines (CXCL8; CCL11; CCL3; CCL4; CCL2; CCL5; CXCL10), pro-inflammatory (IL-1β; IL-6; TNF-α; IL-12; IFN-γ; IL-15; IL-17), and regulatory cytokines (IL-1Ra; IL-4; IL-5; IL-9; IL-10; IL-13) along with growth factors (FGF-basic; PDGF; VEGF; G-CSF; GM-CSF; IL-2; IL-7) were measured in parallel batches carried out by a trained technician using a Bio-Plex 200 System (Hercules, CA, USA) at the flow cytometry facility at Fiocruz Minas. The final concentrations of cord blood and mother serum soluble immune mediators were expressed in pg/mL, according to a five-parameter logistic curve fit regression of standard curves.

### Statistical analysis

Descriptive statistics were carried out using the GraphPad Prism 8.0.2 software (GraphPad Software, San Diego, USA). Data normality was assessed by the Shapiro–Wilk test. Considering the non-parametric distribution of all data sets, multiple comparative analysis among HC and COVID-19 subgroups (Acute, Early, Intermediate, and Late) was carried out by Kruskal–Wallis followed by Dunn’s post-test. The statistical analysis between cord blood and mother serum paired samples was performed by Wilcoxon test. In all cases, statistical significance was considered at p<0.05.

The analysis of fold change magnitude in the soluble immune mediator was assessed as the ratio between serum and cord blood concentrations in COVID-19 subgroups divided by the median levels observed in healthy controls. Additionally, the fold change magnitude in soluble immune mediators was calculated as the ratio of cord blood concentrations divided by the median values observed in mother serum. Fold changes ≤0.8× and ≥1.5× were included in the set of parameters considered for Venn diagram analysis (available at https://bioinformatics.psb.ugent.be/webtools/Venn/) to identify common and selective attributes among subgroups.

Soluble immune mediator signatures were further assessed to chemokines, cytokines, and growth factors from cord blood and mother serum. For this purpose, the original data of soluble mediators, from cord blood and mother serum samples, expressed as continuous variables (pg/mL) were converted into categorical data presented as proportion (%) of subjects with levels above the cut-off values defined as the overall global median concentration of each soluble mediator (CXCL8 = 3.1; CCL11 = 6.9; CCL3 = 0.8; CCL4 = 5.4; CCL2 = 10.2; CCL5 = 244.5; CXCL10 = 43.3; IL-1β=0.2; IL-6 = 0.6; TNF-α=4.0; IL-12 = 0.3; IFN-γ=1.5; IL-15 = 31.8; IL-17 = 1.5; IL-1Ra=98.4; IL-4 = 0.2; IL-5 = 8.3; IL-9 = 2.8; IL-10 = 2.2; IL-13 = 0.6; FGF-basic=1.7; PDGF=12.2; VEGF=8.0; G-CSF=4.1; GM-CSF=0.4; IL-2 = 0.8 and IL-7 = 2.5 pg/mL). The signatures of cord blood, and mother serum soluble immune mediators were analyzed considering the 50^th^ percentile as a gray zone to identify the set of immune mediators with increased levels in each study group and further assemble as ascendant signature profiles.

Integrative networks of cord blood and mother serum immune mediators were built based on Spearman rank correlation analysis. Cross-correlation between immune mediators from cord blood and mother serum compartments was assessed using the Spearman rank tests. Significant correlations (p<0.05) were employed to construct cluster networks (chemokines; pro-inflammatory cytokines; regulatory cytokines, and growth factors) using the open-source Cytoscape software (available at https://cytoscape.org). Comparative analysis among subgroups was carried out considering the number of correlations observed for each soluble mediator cluster and the total number of correlations computed for each subgroup.

Additional analysis of soluble immune mediators was further performed to compare and summarize the major changes observed in the cord blood and mother serum compartments. For this purpose, Z-score normalization (Z-scores = (original value − )/SD) was applied to construct colormaps. The number of soluble mediators with Z-score ≥4 on each compartment was identified and subsidized in comparative analysis among groups.

## Results

### Soluble immune mediators in umbilical cord blood from neonates born to mothers with acute or convalescent COVID-19

**Figure 2:**
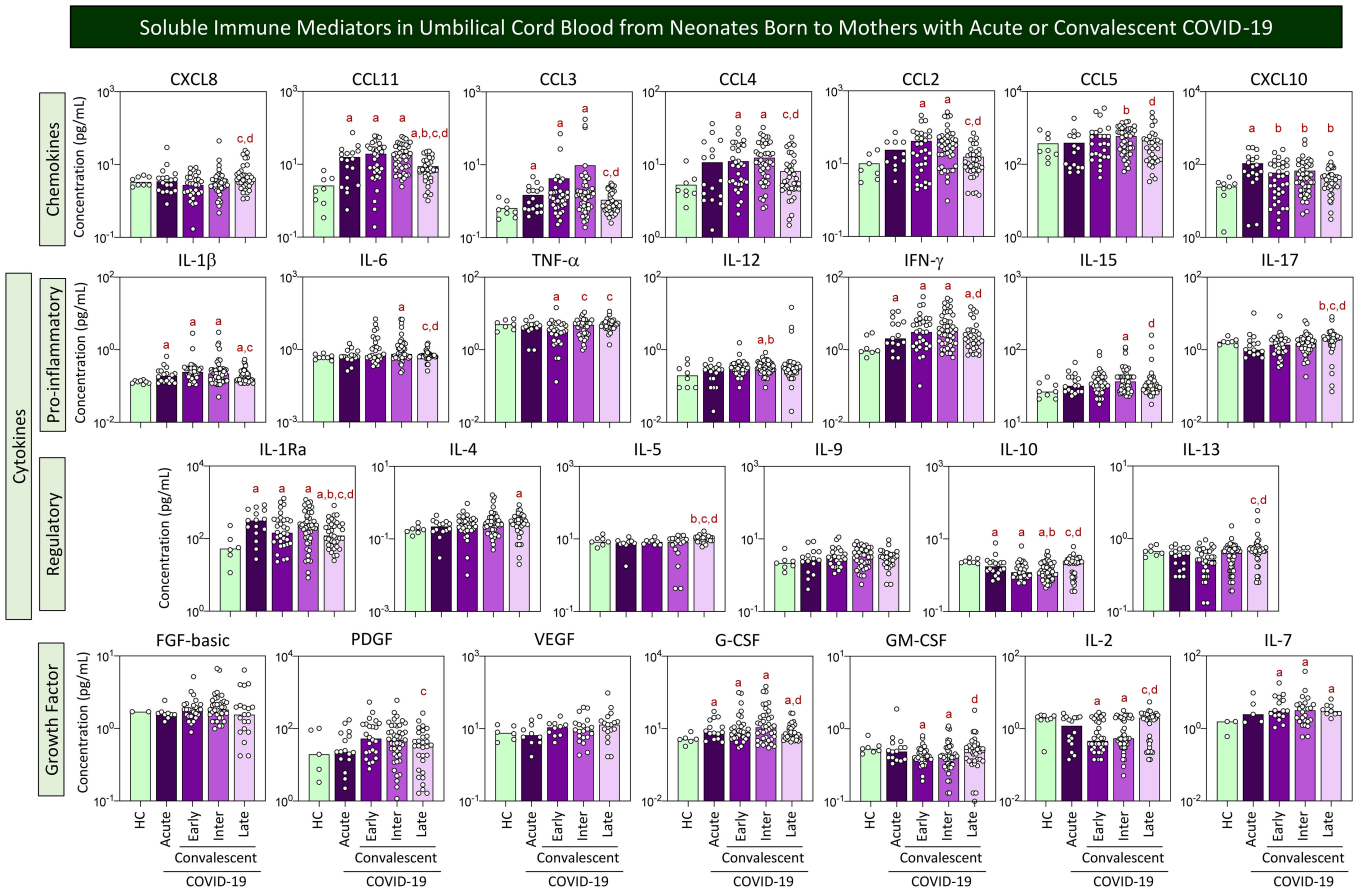
Soluble immune mediators in umbilical cord blood from neonates born to mothers with acute or convalescent COVID-19. The levels of chemokines, pro-inflammatory cytokines, regulatory cytokines, and growth factors were measured in umbilical cord blood samples collected from neonates born to mothers with Acute SARS-CoV-2 infection (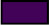, n=18) up to 14 days upon symptom onset or convalescent COVID-19 due to previous SARS-CoV-2 infection, referred to as Early (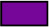, n=34), Intermediate (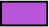, n=50), or Late (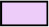, n=39) in comparison with Healthy Controls (HC = 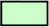, n=8). The levels of soluble immune mediators were quantified by 27-plex high-throughput microbead array as described in Population, Materials and Methods. The results are presented as scattering distribution of individual samples over bar charts showing the median values of cord blood concentration (pg/mL). Multiple comparative analysis was performed by Kruskal–Wallis followed by Dunn’s post-test. In all cases, significance was considered at p<0.05. Significant differences were underscored by the letters “a”, “b”, “c”, “d”, and “e” for comparisons with HC, Acute, Early, Intermediate, and Late groups, respectively.

The levels of chemokines, pro-inflammatory cytokines, regulatory cytokines, and growth factors were measured in umbilical cord blood samples from neonates born to mothers with acute or convalescent COVID-19, and the results are presented in **Figure 2**. Data analysis demonstrated an increase of CCL11, CCL3, CXCL10, IL-1β, IFN-γ, IL-1Ra, and G-CSF and a decrease of IL-10 in cord blood samples from neonates born to mothers with Acute SARS-CoV-2 infection as compared with Healthy Controls (HC). The analysis of cord blood samples at Early and Intermediate convalescent COVID-19 showed increased levels of CCL11, CCL3, CCL4, CCL2, IL-1β, IFN-γ, IL1-Ra, G-CSF, and IL-7 and a decrease of IL-10 and IL-2 as compared with HC. Overall, cord blood samples from the Late convalescent subgroup exhibited few differences as compared with HC, comprising higher levels of CCL11, IL-1β, IFN-γ, IL-4, and IL-7. However, the Late subgroup presented distinct levels of several immune mediators as compared with Early and/or Intermediate subgroups, including increased levels of CXCL8, TNF-α, IL-17, IL-5, IL-10, IL-13, and GM-CSF along with decrease levels of CCL11, CCL3, CCL4, CCL2, CCL5, IL-1β, IL-6, IFN-γ, IL-15, IL-1Ra, PDGF, G-CSF, and IL-2 (**Figure 2**).

### Fold change of soluble immune mediators in umbilical cord blood from neonates born to mothers with acute or convalescent COVID-19 according to healthy controls

**Figure 3:**
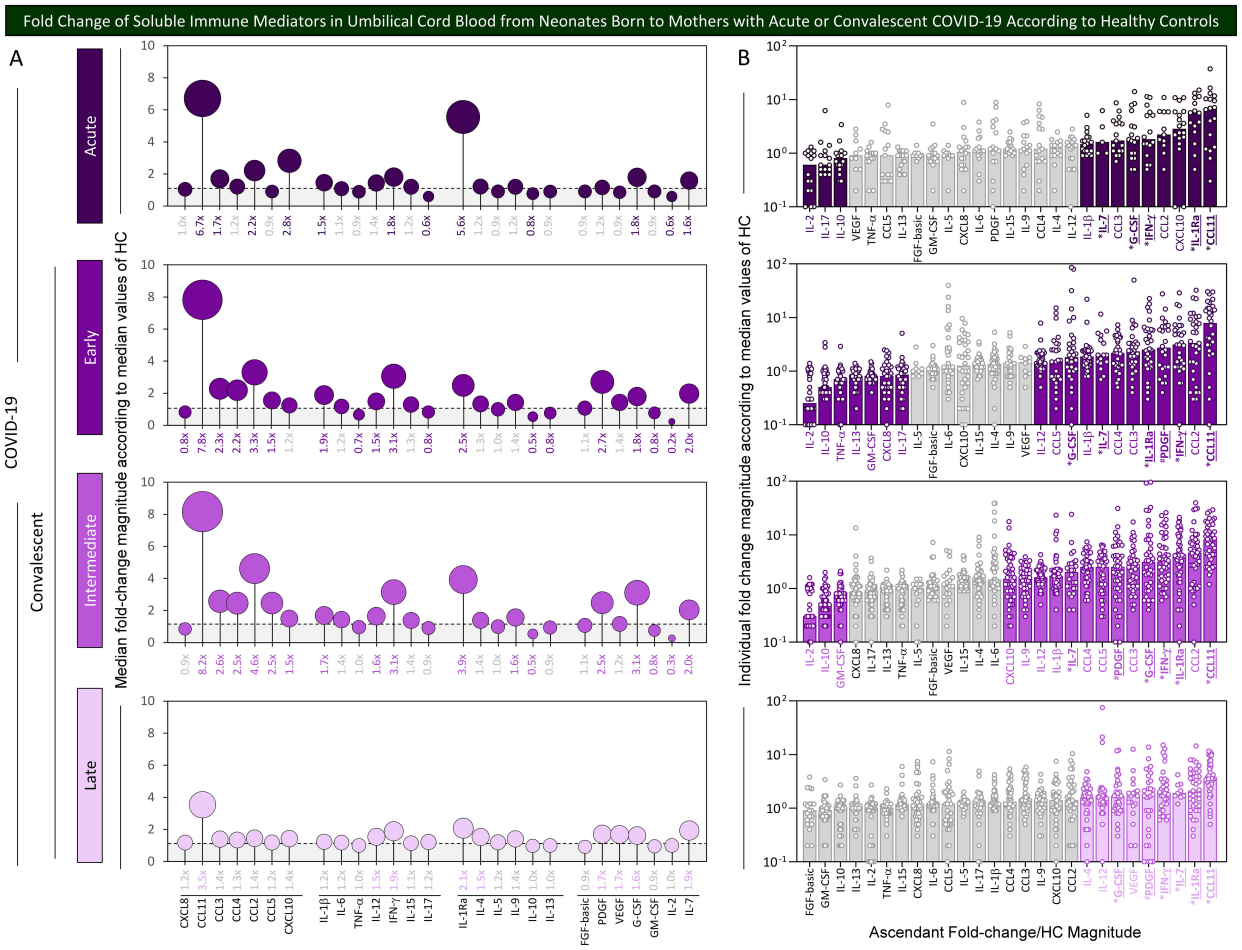
Fold change of soluble immune mediators in umbilical cord blood from neonates born to mothers with acute or convalescent COVID-19 according to healthy controls. The levels of chemokines, pro-inflammatory cytokines, regulatory cytokines, and growth factors were measured in umbilical cord blood samples collected from neonates born to mothers with Acute SARS-CoV-2 infection (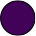, n=18) up to 14 days upon symptom onset or convalescent COVID-19 due to previous SARS-CoV-2 infection, referred to as Early (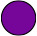, n=34), Intermediate (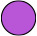, n=50), or Late (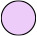, n=39). The levels of soluble immune mediators were quantified by 27-plex high-throughput microbead array as described in Population, Materials and Methods. **(A)** Fold change magnitude is shown in lollipop charts according to the median values observed for Healthy Controls (HC, n=8). Fold change magnitude of soluble immune mediators with significant differences at p<0.05 was underscored by bold underline format. **(B)** The results are shown as scattering distribution of individual fold change/HC median values. Gray color was used to label soluble immune mediators with fold changes (FC) values 0.8x ≤ FC ≥ 1.5x. Common immune mediators observed in all COVID-19 subgroups (Acute, Early, Intermediate, and Late) were labeled with * and those observed only at convalescent subgroups (Early, Intermediate, and Late) identified by ^#^.

Aiming at further characterizing the profile of chemokines, cytokines, and growth factors in umbilical cord blood samples from neonates born to mothers with acute or convalescent COVID-19, the magnitude order of fold changes in soluble immune mediator concentrations was calculated according to HC median values. The results are shown in **Figure 3**. Data analysis demonstrated that cord blood from the Acute subgroup presented a range of soluble immune mediators with fold change magnitudes over 1.5× reaching values as high as 6.7×, with CCL11 > IL1Ra > CXCL10 > CCL2> IFN-γ ~ G-CSF > CCL3 > IL-7 > IL-1β. Cord blood from Early and Intermediate subgroups exhibited a large set of soluble immune mediators with fold change magnitudes higher than 1.5× toward 7.2× and 8.2×, respectively. Overall, data demonstrated a magnitude order of CCL11 > CCL2 > IFN-γ > PDGF > IL-1Ra > CCL3 > CCL4 > IL-7 > IL-1β > G-CSF > CCL5 ~ IL-12 in the Early subgroup and CCL11 > CCL2 > IL-1Ra > IFN-γ ~ G-CSF > CCL3 > PDGF ~ CCL5 ~ CCL4 > IL-7 > IL-1β > IL-12 ~ IL-9 > CXCL10 in the Intermediate subgroup (**Figure 3**, bar charts). Cord blood from the Late subgroup showed a small set of soluble mediators with increased fold changes higher than 1.5× up to 3.5× CCL11 > IL-1Ra > IL-7 ~ IFN-γ > PDGF ~ VEGF > G-CSF > IL-12 ~ IL-4. Conversely, decreased fold change magnitudes below 0.8× were observed in Acute (IL-2 ~ IL-17 < IL-10), Early (IL-2 < IL-10 < TNF-α < IL-13 ~ GM-CSF ~ CXCL8 ~ IL-17), and Intermediate (IL-2 < IL-10 < GM-GSF) with no values below 0.8× observed in the Late subgroup (**Figure 3**, bar charts).

Venn diagram analysis identified a set of common soluble immune mediators with increased levels in all COVID-19 subgroups (from 1.6× up to 8.2×), comprising CCL11, IFN-γ, IL-1Ra, and G-CSF (**Figure 3**, * below bar charts). Moreover, increased fold changes of PDGF were commonly observed only in convalescent COVID-19 (Early, Intermediate, and Late subgroups) (**Figure 3**, ^#^ below bar charts).

### Soluble immune mediator profiles of paired umbilical cord blood samples and serum from mothers with acute or convalescent COVID-19

**Figure 4:**
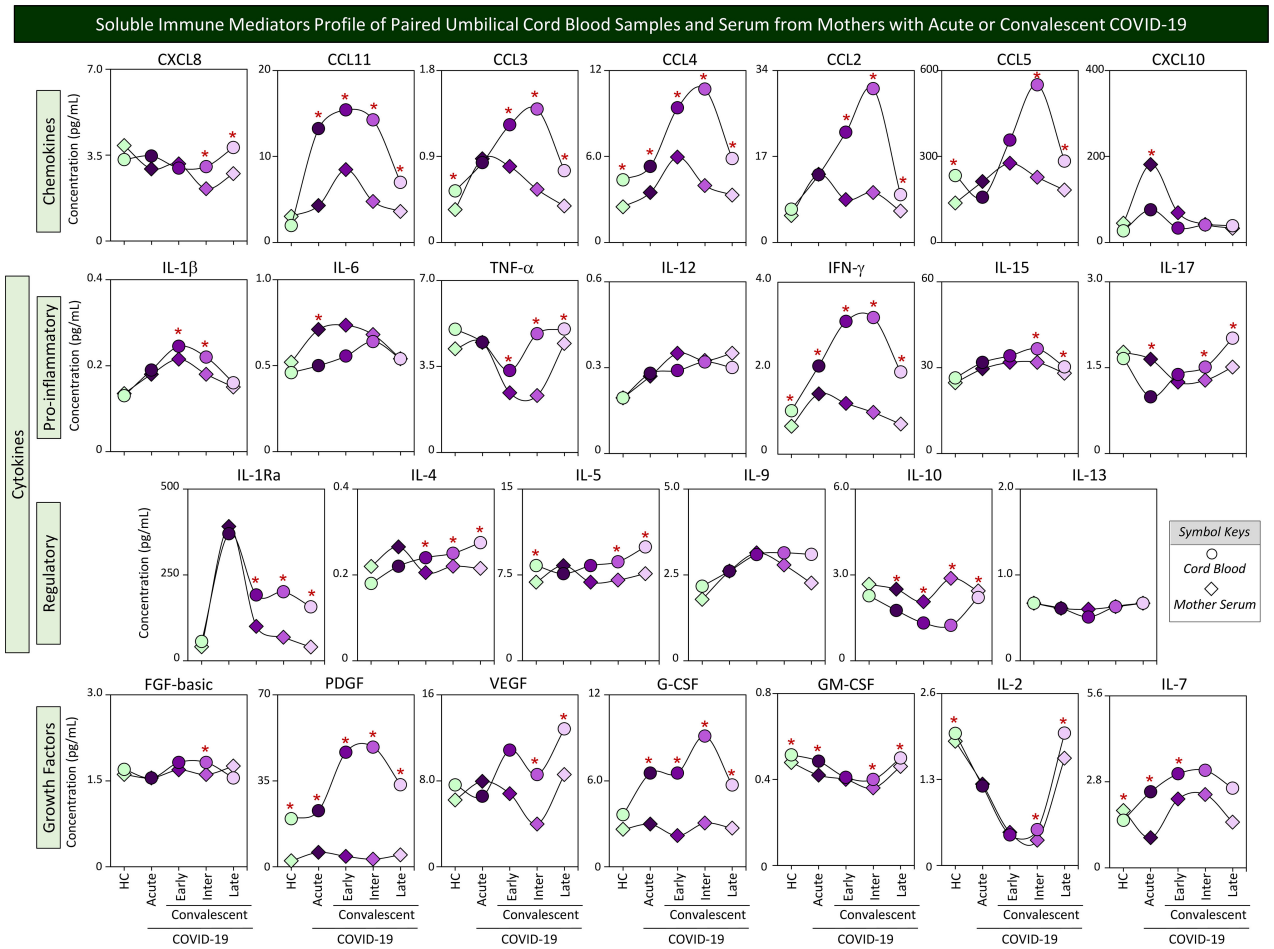
Soluble immune mediator profiles of paired umbilical cord blood samples and serum from mothers with acute or convalescent COVID-19. The levels of chemokines, pro-inflammatory cytokines, regulatory cytokines, and growth factors were measured in umbilical cord blood samples collected from neonates (circles = 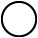) and paired serum samples from mothers (diamonds = 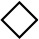) at Acute SARS-CoV-2 infection (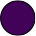;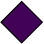, n=18) up to 14 days upon symptom onset or convalescent COVID-19 due to previous SARS-CoV-2 infection, referred as: Early (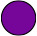;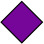, n=34), Intermediate (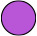;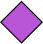, n=50) or Late (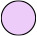;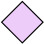, n=39) in comparison with Healthy Controls (HC = 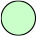;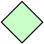, n=8). The levels of soluble immune mediators were quantified by 27-plex high throughput microbead array as described in Population, Materials and Methods. The results are presented as median values of cord blood and mother serum concentration (pg/mL) using symbol keys as shown in the figure. Connecting lines were used to draw soluble immune mediator profiles of cord blood and mother serum samples. Intragroup comparative analysis between paired samples was performed by Wilcoxon test and significance considered at p<0.05. Significant differences were underscored by the red asterisk “*” for comparisons within HC, Acute, Early, Intermediate, and Late groups.

Although transplacental transfer of most soluble mediators is believed to not occur in humans, the maternal immunological profile may interfere in the fetal immune response and impact the cord blood microenvironment. In order to evaluate whether the profile of mother serum immune mediators resembles the pattern observed in cord blood samples from their offsprings, parallel analysis of chemokines, cytokines, and growth factors was performed in mother–newborn pairs from Acute, Early, Intermediate, and Late COVID-19 subgroups as well as healthy controls. The results are presented in **Figure 4**. Data from paired umbilical cord blood (circles) and mother serum samples (diamonds) were compared for each COVID-19 subgroups as well as healthy controls. Connecting lines were used to draw soluble immune mediator profiles. Comparative analysis demonstrated that most soluble immune mediators in mother serum and cord blood paired samples from healthy controls presented similar median values, except for 8 out of 27 molecules (30%), comprising higher levels of CCL3, CCL4, CCL5, IFN-γ, IL-5, PDGF, GM-CSF, and IL-2 and lower levels of IL-7 observed in cord blood as compared with mother serum (**Figure 4**, green symbols). Distinct profiles of soluble immune mediators were identified in the paired analysis of mother–newborn Acute subgroups for 11 out of 27 analytes (41%), comprising increased levels of CCL11, CCL4, IFN-γ, PDGF, G-CSF, GM-CSF, and IL-7 and decreased levels of CXCL10, IL-6, IL-17, and IL-10 in cord blood samples as compared with mother serum (**Figure 4**, dark-purple symbols). Of note were the increased levels of a larger set of soluble immune mediators observed in cord blood samples from convalescent COVID-19 subgroups (Early = 11/27, 41%; Inter = 20/27, 74%; and Late = 18/27, 67%) as compared with mother serum (**Figure 4**). IL-10 was the single parameters presenting lower median levels in cord blood as compared with mother serum throughout convalescent COVID-19 (**Figure 4**).

**Figure 5:**
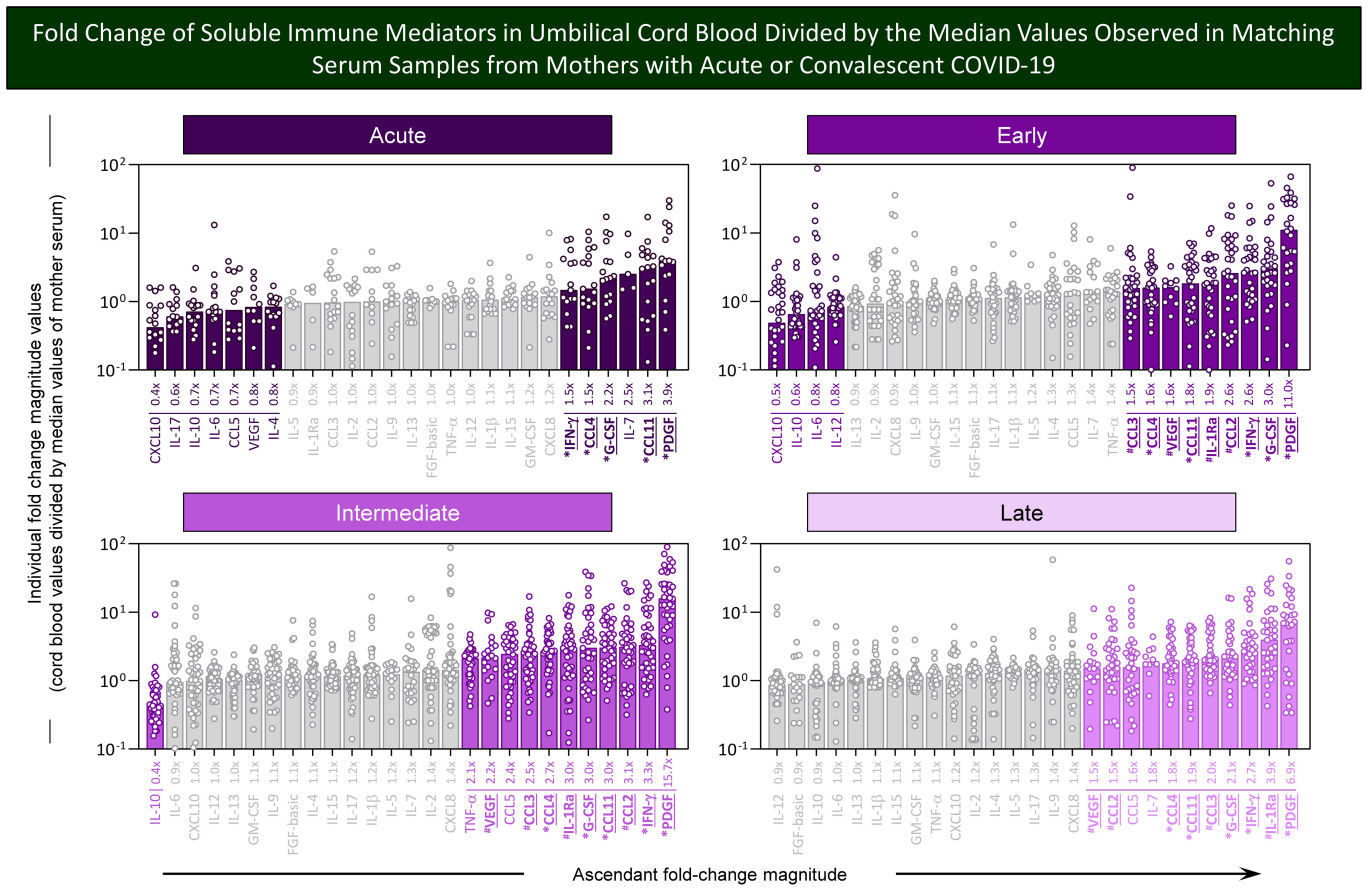
Fold change of soluble immune mediators in umbilical cord blood divided by the median values observed in matching serum samples from mothers with acute or convalescent COVID-19. The levels of chemokines, pro-inflammatory cytokines, regulatory cytokines, and growth factors were measured in umbilical cord blood samples collected from neonates born and paired serum samples from mothers at Acute SARS-CoV-2 infection (n=18) up to 14 days upon symptom onset or convalescent COVID-19 due to previous SARS-CoV-2 infection, referred to as Early (n=34), Intermediate (n=50), or Late (n=39) as well as from Healthy Controls (HC; n=8). The levels of soluble immune mediators were quantified by 27-plex high throughput microbead array as described in Population, Materials and Methods. Fold change magnitude was calculated for umbilical cord blood samples divided by the median values observed for matching mother serum. The results are shown as scattering distribution of individual fold change (cord blood/mother serum median values). Soluble immune mediators with fold changes ≤0.8x and ≥1.5x were labeled by color format. Gray color was used to label soluble immune mediators with fold change (FC) values 0.8x ≤ FC ≥ 1.5x. Common immune mediators observed in all COVID-19 subgroups (Acute, Early, Intermediate, and Late) were labeled with * and those observed only at convalescent subgroups (Early, Intermediate, and Late) identified by ^#^.

Aiming at identifying soluble immune mediators with higher magnitude to differentiate cord blood samples from mother serum, the fold change values were calculated for each soluble immune mediator in cord blood samples from COVID-19 subgroups divided by the median values of matching mother serum. The results are presented in **Figure 5**. Data analysis demonstrated that a range of soluble immune mediators from cord blood from the Acute subgroup presented fold change magnitudes over 1.5× reaching values as high as 3.9×, with PDGF > CCL11 > IL-7 > G-CSF > CCL-4 ~ IFN-γ. Moreover, a larger set of soluble immune mediators from cord blood from convalescent COVID-19 subgroups (Early, Intermediate, and Late) exhibited fold change magnitudes up to 11.0×, 15.7×, and 6.9×, respectively. Overall, data from the Early subgroup demonstrated high fold change magnitude order for PDGF > G-CSF > IFN-γ ~ CCL-2 > IL-1Ra > CCL-11 > VEGF ~CCL4 > CCL3. Data from the Intermediate subgroup showed high fold change magnitude order for PDGF > IFN-γ > CCL2 > CCL11 ~ G-CSF ~ IL-1Ra > CCL4 > CCL3 > CCL5 > VEGF >TNF-α. The results from the Late subgroup showed high fold change magnitude order for PDGF > IL-1Ra > IFN-γ > G-CSF > CCL3 > CCL11 > CCL4 ~ IL-7 > CCL5 > CCL2 ~ VEGF. Conversely, decreased fold change magnitudes (below 0.8x) were observed in Acute (CXCL10 < IL-17 < IL-10 ~IL-6 ~ CCL5 < VEGF < IL-4), Early (CXCL10 < IL-10 < IL-6 ~ IL-12), and Intermediate (IL-10) with no value below 0.8× observed in the Late subgroup (**Figure 5**).

Venn diagram analysis identified a set of common soluble immune mediators with fold change magnitude over 1.5× up to 15.7× in cord blood samples according to mother serum in all COVID-19 subgroups, comprising CCL11, CCL4, IFN-γ, PDFG, and G-CSF (**Figure 5**, * below bar charts). Moreover, increased fold changes of CCL2, CCL3, IL-1Ra, and VEGF were commonly observed only in convalescent COVID-19 (Early, Intermediate, and Late subgroups) (**Figure 5**, ^#^ below bar charts).

### Soluble immune mediator signatures of paired umbilical cord blood samples from neonates and serum from mothers with acute or convalescent COVID-19

**Figure 6:**
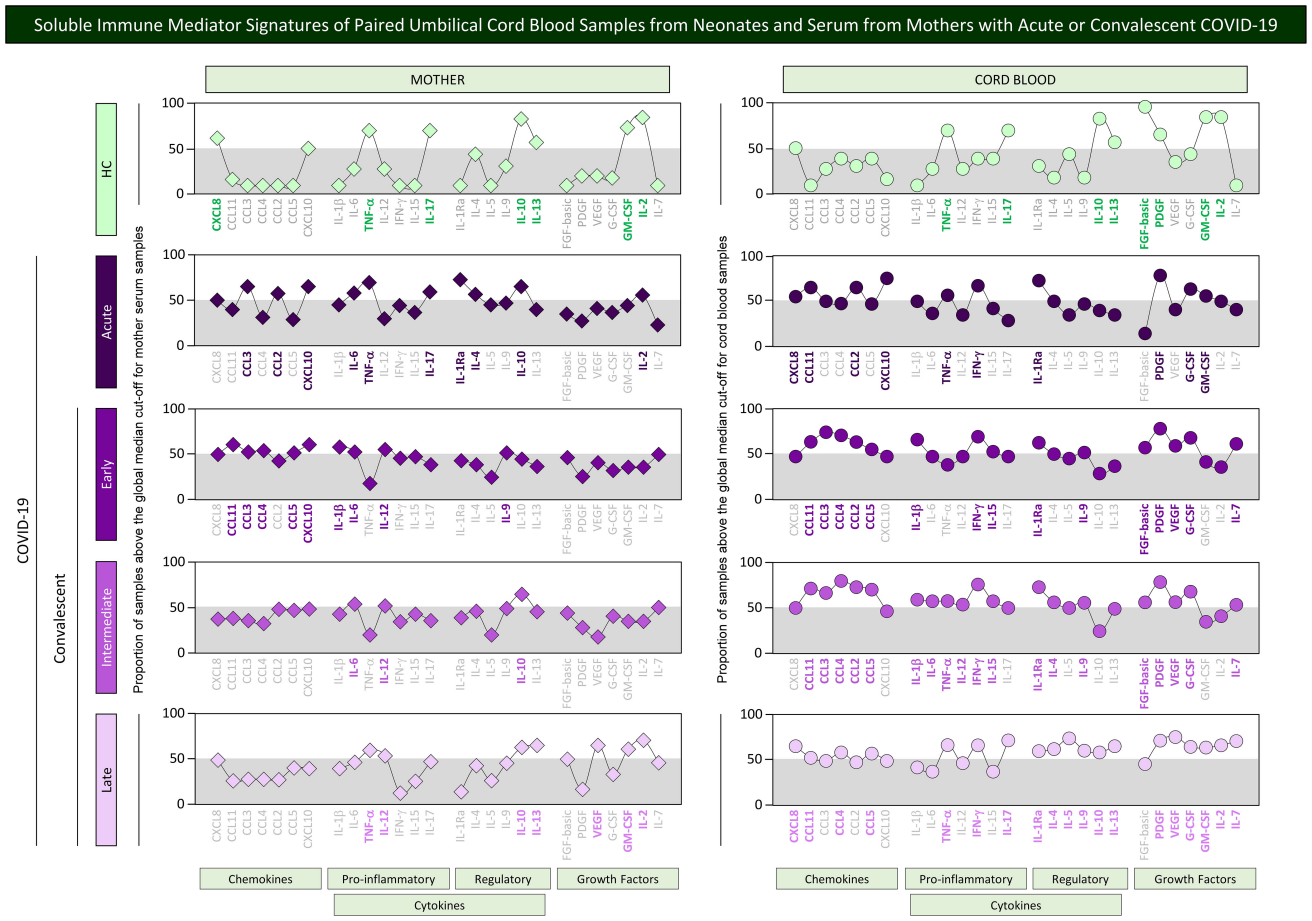
Soluble immune mediator signatures of paired umbilical cord blood samples from neonates and serum from mothers with acute or convalescent COVID-19. The levels of chemokines, pro-inflammatory cytokines, regulatory cytokines, and growth factors were measured in umbilical cord blood samples collected from neonates (circles = 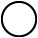) and paired serum samples from mothers (diamonds = 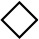) at Acute SARS-CoV-2 infection (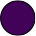;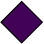, n=18) up to 14 days upon symptoms onset or convalescent COVID-19 due to previous SARS-CoV-2 infection, referred as: Early (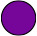;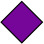, n=34), Intermediate (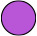;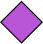, n=50) or Late (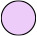;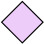, n=39) in comparison with Healthy Controls (HC = 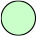;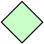, n=8). The levels of soluble immune mediators were quantified by 27-plex high throughput microbead array as described in Population, Materials and Methods. The results are presented in line charts showing the proportion (%) of samples with levels of soluble immune mediators above the cut-off values defined as the overall global median concentration of each soluble mediator from mother serum and cord blood samples as described in Population, Materials and Methods. Connecting lines were used to draw the signatures of distinct categories of soluble immune mediator profiles (chemokines, pro-inflammatory, and regulatory cytokines along with growth factors). The soluble immune mediators with proportion of samples above the 50^th^ percentile (gray zone) were included in the set of attributes with increased levels and were underscored by bold underline color format.

In order to take a panoramic snapshot of soluble immune mediators in mother serum and in the cord blood from their offsprings, the signatures of chemokines, cytokines, and growth factors were assembled for mother–newborn pairs from Acute, Early, Intermediate, and Late subgroups as well as healthy controls. The signatures of soluble immune mediators in mother serum and cord blood were built as categorical data, reported as the proportion of samples with levels above the global median cut-off values determined for mother serum and cord blood samples. The results are presented in **Figure 6**. Signatures from paired mother serum samples (diamonds) and umbilical cord blood (circles) were compared for each COVID-19 subgroups as well as healthy controls. Connecting lines were used to draw the signatures of distinct categories of soluble immune mediator profiles (chemokines, pro-inflammatory and regulatory cytokines, and growth factors). Comparative analysis was carried out to select the set of immune mediators outside the gray zone equivalent to the 50^th^ percentile. In healthy controls, a small set of immune mediators with increased levels were observed for mother serum (7/27, 26%—CXCL8, TNF-α, IL-17, IL-10, IL-13, GM-CSF, IL-2) and cord blood samples (8/27, 30%—TNF-α, IL-17, IL-10, IL-13, FGF-basic, PDGF, GM-CSF, IL-2). In the Acute subgroup, a total of 10 out of 27 immune mediators (37%) were increased in mother serum (CCL3, CCL2, CXCL10, IL-6, TNF-α, IL-17, IL-1Ra, IL-4, IL-10, and IL-2) and cord blood samples (CXCL8, CCL11, CCL2, CXCL10, TNF-α, IFN-γ, IL-1Ra, PDGF, G-CSF, and GM-CSF) (**Figure 6**).

Distinct profiles of soluble mediators were identified in mother serum and cord blood from convalescent subgroups. Waning in the set of immune mediators was observed for mother serum from the Early (9/27, 33%), Intermediate (3/27, 11%), and Late subgroups (7/27, 26%). On the other hand, a higher number of immune mediators remained with increased levels in cord blood samples in the convalescent subgroups (Early = 15/27, 56%; Intermediate = 19/27, 70%, and Late = 19/27, 70%) (**Figure 6**).

**Figure 7:**
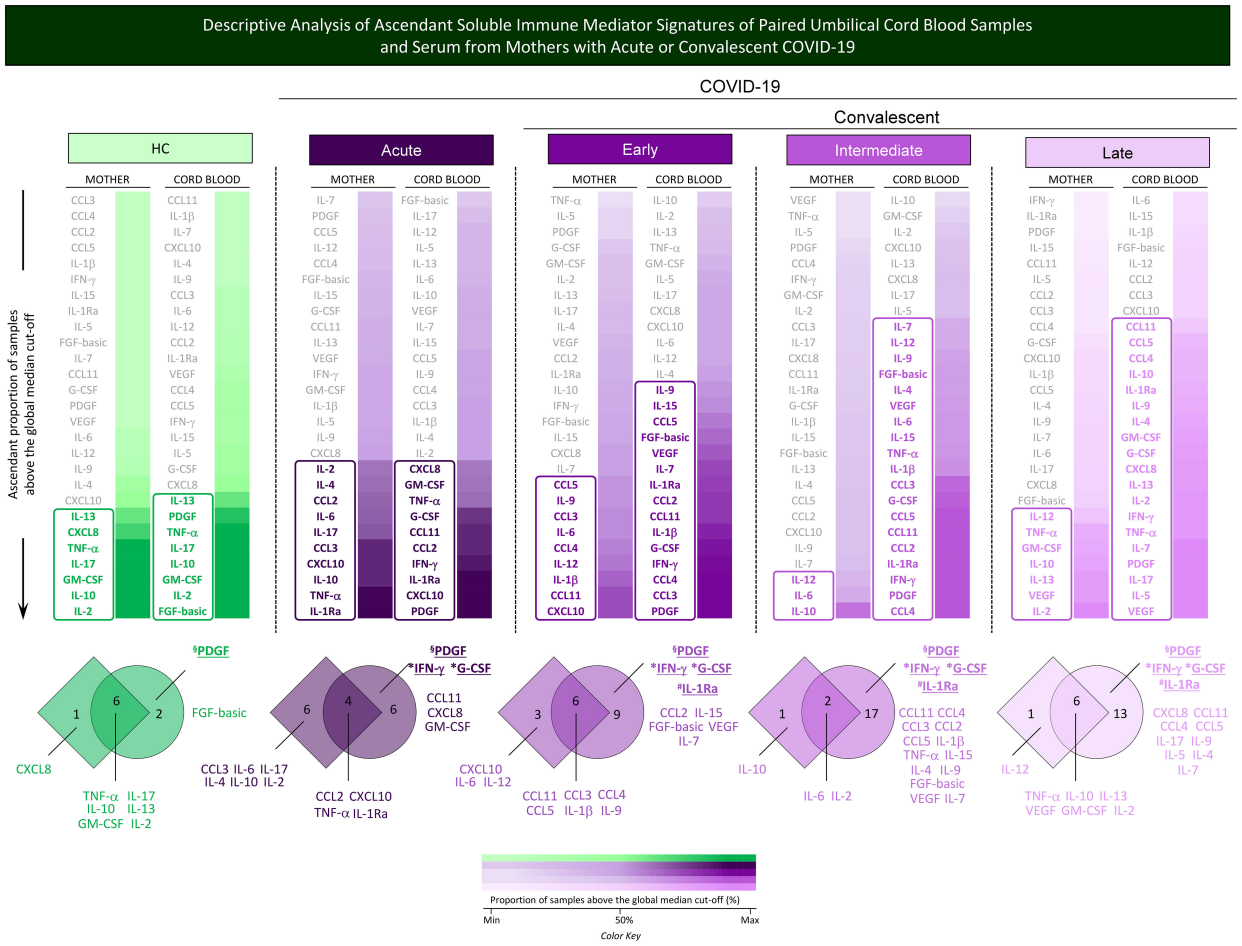
Descriptive analysis of ascendant soluble immune mediator signatures of paired umbilical cord blood samples and serum from mothers with acute or convalescent COVID-19. The levels of chemokines (CXCL8, CCL11, CCL3, CCL4, CCL2, CCL5, CXCL10), pro-inflammatory cytokines (IL-1β, IL-6, TNF-α, IL-12, IFN-γ, IL-15, IL -17), regulatory cytokines (IL-1Ra, IL-4, IL-5, IL-9, IL-10, IL-13), and growth factors (FGF-basic, PDGF, VEGF, G-CSF, GM- CSF, IL-2, IL-7) were measured in umbilical cord blood samples collected from neonates and serum samples from mothers at Acute SARS-CoV-2 infection (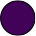;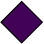, n=18) up to 14 days upon symptom onset or convalescent COVID-19 due to previous SARS-CoV-2 infection, referred to as Early (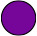;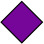, n=34), Intermediate (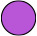;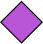, n=50), or Late (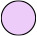;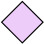, n=39) in comparison with Healthy Controls (HC = 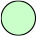;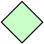, n=8). The levels of soluble immune mediators were quantified by 27-plex high-throughput microbead array as described in Population, Materials and Methods. The results as ascendant profile of the proportion (%) of samples with levels of soluble immune mediators above the cut-off values, defined as the overall global median concentration of each soluble mediator as described in Population, Materials and Methods. Colormaps were created to identify the soluble immune mediators displaying a proportion of subjects above 50% and select the set of attributes with increased levels (column rectangles), underscored by bold color format. The color key used for number proportion (%) of samples with levels of soluble immune mediators above the cut-off values (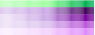; Min, 50% and Max) is provided in the figure. Venn diagrams were constructed to identify the set of common and selective attributes observed in cord blood and mother serum samples within the HC, Acute, Early, Intermediate, and Late groups. The common immune mediator observed in all groups (HC, Acute, Early, Intermediate, and Late) was tagged by §; those commonly observed in all COVID-19 subgroups (Acute, Early, Intermediate, and Late) were labeled with * and those observed only at convalescent subgroups (Early, Intermediate, and Late) identified by ^#^.

Additional analysis of soluble immune mediator signatures was performed to identify common and selective molecules with increased levels in mother serum and cord blood pairs in healthy controls and during acute or convalescent COVID-19. For this purpose, the soluble immune mediators were ranked considering the ascendant proportion of samples with levels above the global median and data presented in **Figure 7**. Venn diagram analysis allowed the identification of common and selective immune mediators observed in mother–newborn pairs. Descriptive analysis of selective immune mediators pointed out that PDGF was universally increased in cord blood samples from all study groups (HC, Acute, Early, Intermediate, and Late). IFN-γ and G-CSF were increased in cord blood samples of all COVID-19 subgroups (**Figure 7**, * underscored attributes). IL-1Ra was selectively increased in cord blood from convalescent subgroups (Early; Intermediate; Late) (**Figure 7**, ^#^ underscored attributes).

### Integrative networks of soluble immune mediators in paired umbilical cord blood samples from neonates and serum from mothers with acute or convalescent COVID-19

**Figure 8:**
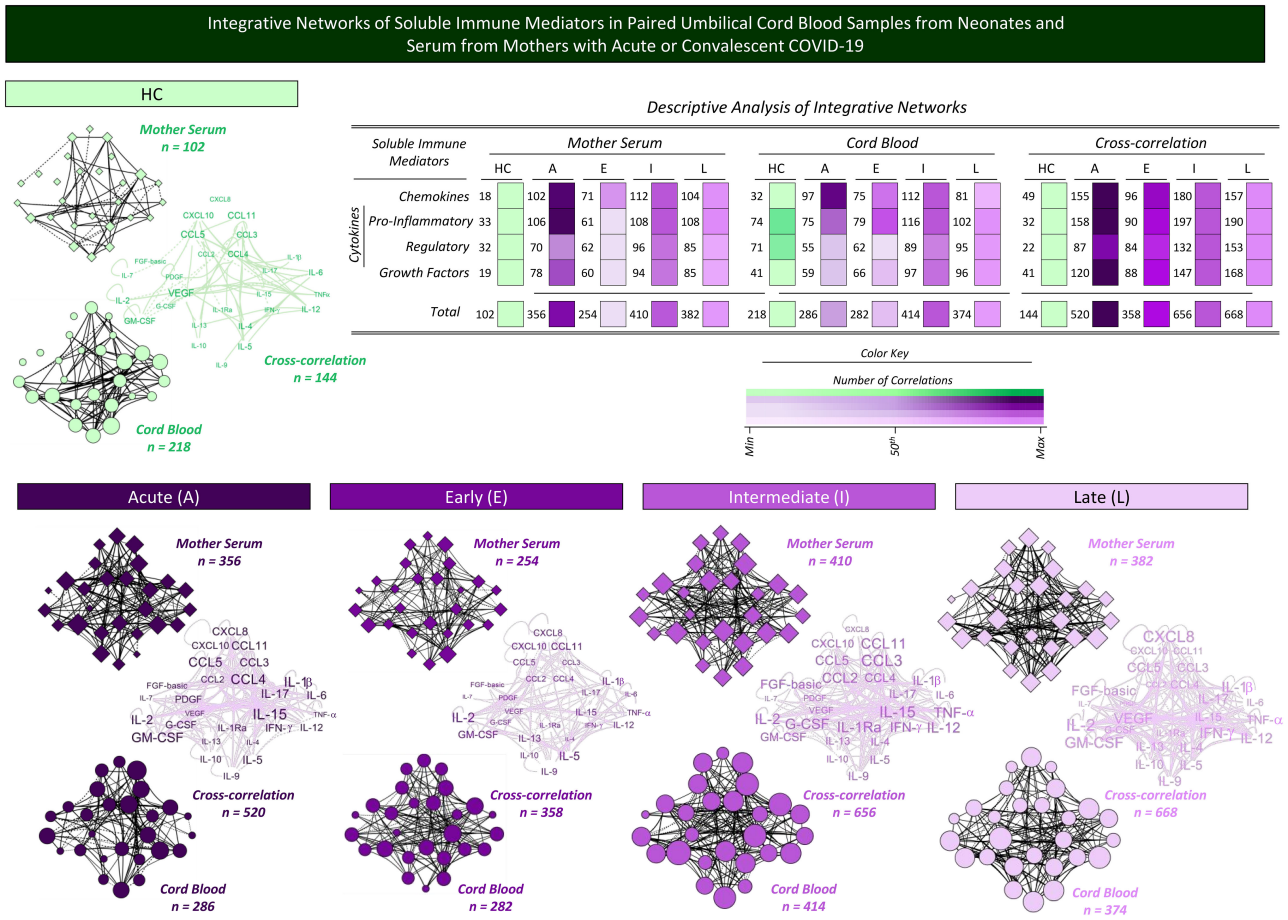
Integrative networks of soluble immune mediators in paired umbilical cord blood samples from neonates and serum from mothers with acute or convalescent COVID-19. Integrative network circuits were assembled for serum chemokines, pro-inflammatory cytokines, regulatory cytokines, and growth factors measured in cord blood samples collected from neonates (circles = 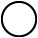) and paired serum samples from mothers (diamonds = 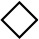) at Acute SARS-CoV-2 infection (A = 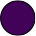;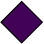, n=18) up to 14 days upon symptom onset or convalescent COVID-19 due to previous SARS-CoV-2 infection, referred to as Early (E = 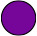;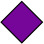, n=34), Intermediate (I = 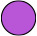;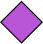, n=50), or Late (L = 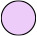;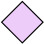, n=39) in comparison with Healthy Controls (HC = 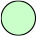;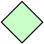, n=8). The levels of soluble immune mediators were quantified by 27-plex high-throughput microbead array as described in Population, Materials and Methods. The network circuits were built based on Spearman rank test correlation analysis between pairs of soluble immune mediators. Only moderate/strong (“r” scores ≥ |0.40|) significant correlations (p<0.05) were used to build networks with four cluster circuits (chemokines, pro-inflammatory cytokines, regulatory cytokines, and growth factors) using the open-source Cytoscape software platform (available at https://cytoscape.org). Cross-correlation clouds were assembled to compile the correlograms of soluble immune mediators from mother serum and cord blood samples. The number of correlations within each cluster and the total correlation of soluble immune mediators for each group are provided in the inserted table. Color maps illustrate the comparative analysis of correlation numbers within each cluster among subgroups from mother serum, cord blood samples, and their cross-correlation. The color key used for number of correlations (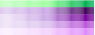; Min, 50^th^, and Max) is provided in the figure.

A complementary landscape of interplay between soluble immune mediators from mother serum and cord blood samples was explored by correlation and cross-correlation analysis. The results are presented in **Figure 8**. Correlation analysis between chemokines, cytokines, and growth factors was performed to assess the neighborhood connectivity in mother serum and cord blood microenvironments. Additionally, cross-correlation analysis between mother serum and cord blood soluble mediators was calculated to characterize the interplay of soluble immune mediators between these compartments. Data analysis demonstrated that overall, the number of correlations in mother serum and cord blood, as well as the cross-correlation between mother/cord blood pairs, increased in Acute COVID-19 as compared with HC (n=102 and 356; n=218 and 286; n=144 and 520, respectively). Increased correlation numbers in cord blood (n=414 and 374) along with higher cross-correlation between mother/cord blood pairs (n=656 and 668) were observed from acute infection toward Intermediate and Late convalescent subgroups (**Figure 8**).

### Summary of changes in soluble immune mediators in paired umbilical cord blood samples and serum from mothers with acute or convalescent COVID-19

**Figure 9:**
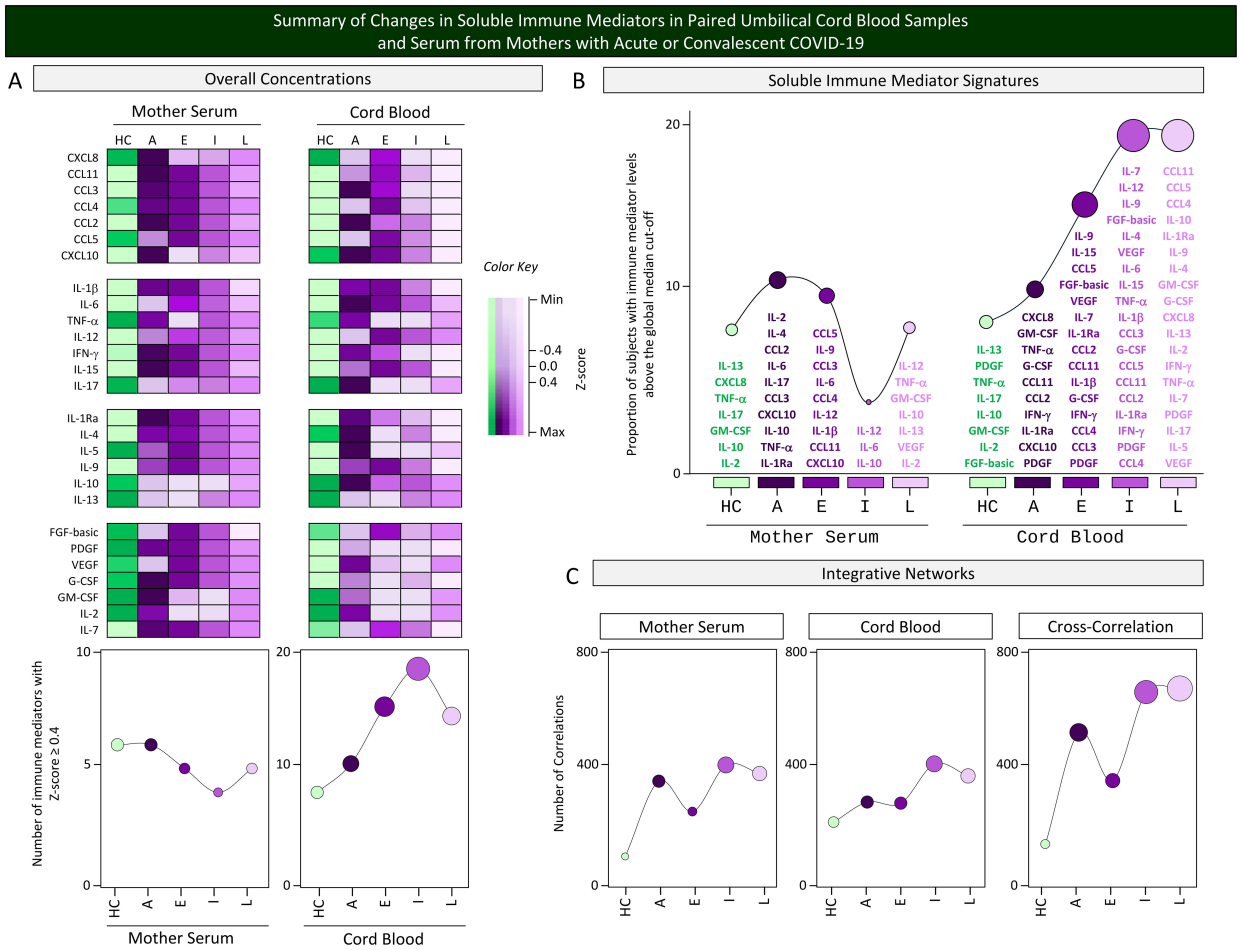
Summary of changes in soluble immune mediators in paired umbilical cord blood samples and serum from mothers with acute or convalescent COVID-19. A snapshot of **(A)** the overall concentrations, **(B)** soluble immune mediator signatures, and **(C)** integrative networks of soluble immune mediators (chemokines, pro-inflammatory cytokines, regulatory cytokines, and growth factors) was composed umbilical cord blood samples collected from neonates and serum samples from mothers at Acute SARS-CoV-2 infection (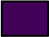, n=18) up to 14 days upon symptom onset or convalescent COVID-19 due to previous SARS-CoV-2 infection, referred to as Early (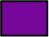, n=34), Intermediate (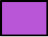, n=50), or Late (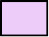, n=39) in comparison with Healthy Controls (HC = 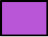, n=8). To summarize the major changes observed in the overall concentration of soluble mediators in cord blood and mother serum compartments, Z-score normalization was applied and colormaps constructed to calculate the number of soluble mediators with Z-score ≥4 on each compartment. The results are presented in line charts to illustrate the comparative analysis among subgroups. The soluble immune mediator signatures were compiled to allow comparisons between the sets of soluble mediators with increased levels in cord blood and mother serum samples from each subgroup. Line charts illustrate the rhythm of immune mediators. Integrative networks were compared considering the number of correlations observed for cord blood, mother serum, and the cross-correlation between them in each subgroup. Line charts illustrate the profile of correlation between immune mediators in each subgroup.

A panoramic snapshot of changes in the overall concentrations, signatures, and integrative networks of soluble immune mediators was taken for comparative analysis between cord blood samples and serum from mothers with acute or convalescent COVID-19. The results are presented in **Figure 9**. Colormap constructs were assembled for comparative analysis of soluble immune mediator concentrations between the two compartments. Data demonstrated an inverted profile for mother serum and cord blood samples. While a decline in soluble mediator was observed in the mother serum compartment from acute toward convalescent COVID-19 subgroups, an increase of soluble mediators was observed in cord blood samples from acute to convalescent COVID-19. The analysis of soluble mediator signatures further confirmed these findings. An increase in correlation numbers was observed in mother serum and cord blood samples and in the cross-correlation interplay from HC toward Late convalescent COVID-19. However, the waveforms revealed that during acute infection, a more pronounced upregulation was observed in the correlation numbers in mother serum and in the cross-correlation interplay as compared with cord blood samples (**Figure 9**).

## Discussion

The emergence of newly identified pathogens or previously known pathogens that are spreading to new geographic areas or populations presents significant challenges to global health. The understanding of the pathogenesis of these emerging infectious diseases, particularly in a vulnerable population such as pregnant women, besides the identification of potential biomarkers in the maternal–fetal interface are extremely important for proposing clinical interventions. The present study attempts to characterize the profile of soluble immune mediators in serum samples from mother with acute and convalescent COVID-19 and in the umbilical cord blood microenvironment of their offsprings, in comparison to healthy controls, employing distinct statistical approaches for data analysis. The unique aspect of this study lies in the measurement of immune mediator signatures in mother/newborn dyads and the observation that these signatures differ significantly, highlighting the importance of evaluating distinct microenvironments independently while monitoring COVID-19-affected pregnancies.

The understanding of the immune response to SARS-CoV-2 infection has rapidly expanded from extensive basic research toward clinical applications (16–18). The scientific literature on COVID-19 immunity during pregnancy remains limited (4, 19, 20). The physiological changes in the immune system observed during pregnancy make the pregnant women and theirs offsprings a risk group for adverse outcomes of SARS-CoV-2 infection (1, 6–8). Although the maternal–fetal transmission of the SARS-CoV-2 infection is rare, the immunological changes during pregnancy can increase the risk for complications in COVID-19-affected pregnancies (21, 22).

The interplay between the maternal–fetal interface comprises complex physiological changes and undergoes modifications along the pregnancy trimesters (11). Therefore, the analysis of the maternal–fetal interface during acute and convalescent COVID-19 is relevant for assessing the dynamics of changes in the immune responses of mothers and the impact in the umbilical cord blood microenvironment. Regardless of the relevance of this topic, the effect of SARS-CoV-2 during pregnancy, as well as the long-term impact on infant development, remains to be elucidated.

In this line, the present study aimed to investigate whether the profile of soluble immune mediators in cord blood mirrors that observed in serum from mothers with acute (at delivery) and Early/Intermediate/Late convalescent SARS-CoV-2 infection (3^rd^, 2^nd^, and 1^st^ Trimesters, respectively). For this purpose, paired samples of mother serum and umbilical cord blood from their neonates were obtained at delivery and categorized according to the time SARS-CoV-2 infection during pregnancy. It has been shown that perinatal acquired viral infections may lead to relevant neonatal complications, and the pregnancy trimester of SARS-CoV-2 infection is crucial to determining the risk of adverse outcomes (23–26).

Our results demonstrated that during Acute infection, cord blood samples presented an increase of several soluble immune mediators, including CCL11, CCL3, CXCL10, IL-1β, IFN-γ, IL-1Ra, and G-CSF and a decrease of IL-10, resembling the classically reported cytokine storm elicited by SARS-CoV-2 infection. The upregulation in CXCL10 has been previously reported during SARS-CoV-2 infection acquired at the 3^rd^ pregnancy trimester, aligning with our observation (27, 28).

Our findings showed that as maternal infection progresses from acute toward the convalescent phase, the changes in cord blood immune mediators did not mirror the mother serum profile. These findings suggested that the impact of SARS-CoV-2 infection during pregnancy triggers compartmentalized changes in the immunological profile of mother serum and cord blood, with a long-term effect in the latter contrasting with the typical waning observed for mother serum. Some studies have reported the endothelial dysfunction and placental vascular flow changes due to COVID-19 infection favoring the passage of soluble mediators and other components of the immune system into the cord blood (29, 30). The precise mechanism underlying the crosstalk between systemic and compartmentalized microenvironments, such as mother serum and cord blood dyad, as well as mother serum and cerebrospinal fluid, still remains to be elucidated. In this context, the existence of a divergent landscape of soluble immune mediators in serum and cerebrospinal fluid has been demonstrated, emphasizing the relevance of understanding the systemic and compartmentalized immune response elicited by SARS-CoV-2 infection during pregnancy (31). Therefore, the immunological response during pregnancy is the result of the combination and specificities from the maternal immune system and the fetal–placental microenvironment, leading to a unique immunological response (32).

The COVID-19 infection during pregnancy elicits a maternal systemic inflammatory response leading to changes in the placenta microenvironment. These changes can incite an immune dysregulation in the fetus that ultimately portrays short- and long-term effects in offspring (33). Our findings support the hypothesis of active fetal–placental participation in immune responses. As demonstrated by Taglauer and colleagues (27), whereas some cytokines and chemokines were elevated in the neonate compartment, their presence was not simply a result of passive transfer from maternal circulation. These findings align with previous observations that changes in the cord blood immune mediators do not represent a passive transfer from mother serum but rather indicate independent mechanisms that occur within the placental–fetal dyad (11, 27).

The analysis of the mother–cord blood dyad allowed the characterization of the immune response dialog in the maternal–fetal interface across acute and convalescent COVID-19 acquired during pregnancy. Regardless of the COVID-19 stage, cord blood samples from neonates born to mothers with COVID-19 exhibited higher levels of CCL11, IFN-γ, IL-1Ra, and G-CSF as compared with healthy controls. Of note, it is important to mention that CCL4 displayed an increase in convalescent subgroups, and PDGF was also increased in cord blood among all the subgroups, including HC. These data are consistent with the known effects of SARS-CoV-2, which can trigger a polyfunctional systemic immune activation even in neonatal compartments. Moreover, these findings are in agreement with the hypothesis that changes in the cord blood immune mediators represent an independent mechanism within the placental–fetal interface (11, 27, 34). In this context, this phenomenon, referred to as utero priming, even in the absence of congenital bacterial and virus infection, is characterized by changes in fetal cytokines, chemokines, and other cellular and humoral factors that do not resemble the maternal response (35, 36). The knowledge of this distinct fetal microenvironment as compared with the maternal immune response brings about novel insights regarding the compartmentalized immune response induced by gestational COVID-19.

Previous studies have demonstrated that SARS-CoV-2 infection during pregnancy induces a proinflammatory state within the amniotic cavity, causing an increase of immune mediators in fetal circulation (37). Our data highlighted the presence of elevated CCL11 and IFN-γ levels in cord blood samples that may indicate a heightened state of inflammation at the maternal–fetal interface. Moreover, the higher levels of IL-1Ra, an anti-inflammatory molecule, may play a role as a compensatory mechanism to counteract the pro-inflammatory environment induced by maternal COVID-19, protecting the neonate compartments from excessive inflammation that could disrupt development (38). Previous studies have reported that increased levels of IL-1Ra during SARS-CoV-2 infection may predict patient severe COVID-19 outcomes and might be useful as a prognostic biomarker to guide treatment strategies (39).

The persistence of G-CSF in cord blood samples, due to the acute to convalescent phases, further underscore the complexity of the immune response at the maternal–fetal interface. G-CSF is known to play a role in both immune defense and tissue repair, suggesting that its elevation may reflect an attempt to balance immune activation with tissue homeostasis (38, 40). It is well known that G-CSF increases during pregnancy, encompassing the maps of cytokine landscapes of distinct phases of gestational immune response (11). It has been suggested that G-CSF plays a role as an anti-inflammatory mediator during pregnancy (11).

Our data demonstrated higher levels of CCL4, particularly in the convalescent subgroups, suggesting persistent immune activation even after the resolution of acute infection. CCL4, a chemokine involved in the recruitment of monocytes and lymphocytes, may contribute to the long-term immunological response and some reports that identify this chemokine as a redundant molecule in correlations between other immune system components in the maternal–fetal interface (41). In addition, all cord blood subgroups displayed higher levels of PDGF, regardless of the SARS-CoV-2 maternal infection. In this context, the role of this molecule can reflect a baseline physiological process that is essential for fetal development. However, its specific role in the context of maternal COVID-19 and the maternal–fetal interface along with their crossing to the fetus compartments warrants further investigation. These observations can reflect a complex and persistent maternal–fetal interface even during the convalescent phase of COVID-19, suggesting that the effects of maternal infection may extend beyond the acute phase, potentially influencing neonatal and childhood health outcomes. Some reports have shown that maternal viral infections, such as influenza and cytomegalovirus, can lead to persistent changes in fetal cytokine profiles, with potential long-term consequences for immune function and disease susceptibility (24). In addition, this type of immunity transfer, called protective immunity, refers to the gradual priming of the fetal immune system for adaptive memory development *in utero*, and there is evidence that fetal inflammation may also be beneficial for immune priming (36).

The comparative analysis of immune mediators in the mother–newborn dyad demonstrated that certain mediators, such as PDGF and G-CSF, were markedly elevated in cord blood relative to maternal serum, especially during convalescence, with fold changes up to 15.7×. This differential pattern between maternal serum and cord blood suggests a distinct immune response in the fetal environment compared with the maternal circulation. Integrative network analysis further revealed increased neighborhood connectivity among immune mediators in both microenvironments, particularly in the context of late convalescent COVID-19. It has been postulated that immune response networks can integrate pathways by the engagement of three types of interactions, including cooperation, complementation, and compensation (42). These interactions are relevant to guarantee the redundancy of immune mediator pathways that ensure the robustness of the immune system. In this line, in the present study, we have explored the concept of redundancy and robustness of the immune system using the concept of integrative networks, assembled according to the number of correlations between immune mediators from mother serum and cord blood that can be transposed to the principles of cooperation, complementation, and compensation among functional properties of immune mediators. The analysis of immune mediator interactions is relevant for better understanding the immunological changes associated with SARS-CoV-2 infection during distinct gestational phases. Our data demonstrated that soluble immune mediators in mother–newborn pairs showed an increase of neighborhood connectivity both in microenvironments and in their interplay from acute toward late convalescent COVID-19. We believe that a reasonable biological interpretation for these findings is that SARS-CoV-2 infection acquired in the first pregnancy trimester led to an increased redundancy and robustness of immune interactions in the mother/newborn dyad. As the immune system gains redundancy, the establishment of additional compensatory mechanisms is observed. In this sense, the analysis of correlations between immune mediators and their variation represents a rational approach to characterize the compartmentalized immune response in the cord blood and mother serum microenvironments. Our findings pave the way for future investigations into the role of specific immunological mediators in modulating neonatal immunity and their impact on child development.

The present study has some limitations. In spite of the large number of mother–newborn paired samples analyzed in this exploratory investigation, the described observational design with multiple comparisons without corrections for comorbidities, clinical manifestations, disease severity, or other confounding variables constituted a study limitation. As the present study was carried out during the circulation of B.1.1.28 and B.1.1.33 SARS-CoV-2 strains, the generalization of our findings to epidemiological scenarios of other SARS-CoV-2 variants is limited since distinct variants of concern may interfere in the immune response. Another limitation is that the present investigation is a single-center study. Moreover, we have not performed a prospective follow-up analysis of the children to monitor their immune response profile.

In summary, this pioneering study investigated the complex immunological interaction between mothers with acute and convalescent COVID-19 and their newborns, revealing distinct immunological signatures in maternal serum and umbilical cord blood. Our findings demonstrated distinct profiles in cord blood as compared with mother serum, bringing about novel insights regarding the potential impact of maternal SARS-CoV-2 infection at distinct gestational phases on neonatal health and development. While acute SARS-CoV-2 infection in the mother induces a systemic inflammatory response in both compartments, the convalescent phase presents a more differentiated immunological pattern in umbilical cord blood, suggesting a long-term effect on the fetal environment. The persistent elevation of mediators such as CCL11, IFN-γ, IL-1Ra, and G-CSF in umbilical cord blood, regardless of the stage of maternal COVID-19, underscores the importance of “*in utero* priming” and its potential to modulate neonatal immunity in the long term. The striking differences in the levels of mediators such as PDGF and G-CSF between maternal serum and umbilical cord blood, especially during convalescence, reinforce the need to evaluate the maternal–fetal environment as distinct immunological compartments. Although this study provides valuable insights into the maternal–fetal immune response to SARS-CoV-2 infection, we acknowledge the limitations inherent in its observational design and the specificity of the SARS-CoV-2 variants circulating during data collection. Future studies should focus on longitudinal analyses, with greater sample diversity, to monitor the long-term effects of maternal SARS-CoV-2 exposure on child health. Ultimately, a detailed understanding of the maternal–fetal immunological interface in response to COVID-19 is crucial for developing effective prevention and intervention strategies, aiming to protect the health of mothers and newborns during and after the pandemic. Together, this evidence regarding the maternal–fetal crosstalk can subsidize the improvement of clinical practice and public health policies for management of prenatal exposure to SARS-CoV-2 infection.

## Data Availability

The raw data supporting the conclusions of this article will be made available by the authors, without undue reservation.
